# Electrospray mode transition of microdroplets with semiconductor nanoparticle suspension

**DOI:** 10.1038/s41598-017-05175-6

**Published:** 2017-07-11

**Authors:** Eduardo Castillo-Orozco, Aravinda Kar, Ranganathan Kumar

**Affiliations:** 1 Department of Mechanical and Aerospace Engineering, University of Central Florida, Orlando, Florida USA; 2 CREOL, The College of Optics and Photonics, University of Central Florida, Orlando, Florida USA

## Abstract

Electrosprays operate in several modes depending on the flow rate and electric potential. This allows the deposition of droplets containing nanoparticles into discrete nanodot arrays to fabricate various electronic devices. In this study, seven different suspensions with varying properties were investigated. In the dripping mode, the normalized dropsize decreases linearly with electric capillary number, *Ca*_*e*_, (ratio of electric to surface tension forces) up to *Ca*_*e*_ ≈ 1.0. The effect of viscous forces is found to be negligible in the dripping mode since the capillary number is small. For flow rates with low Reynolds number, the mode changes to microdripping mode, and then to a planar oscillating microdripping mode as *Ca*_*e*_ increases. The normalized dropsize remains nearly constant at 0.07 for *Ca*_*e*_ > 3.3. The microdripping mode which is important for depositing discrete array of nanodots is found to occur in the range, 2 ≤ *Ca*_*e*_ ≤ 2.5. The droplet frequency increases steadily from dripping to microdripping mode, but stays roughly constant in the oscillating microdripping mode. This work provides a physical basis by which the flow rate and the voltage can be chosen for any nanosuspension to precisely operate in the microdripping mode at a predetermined dropsize and droplet frequency.

## Introduction

Nanoparticles of various materials are known to exhibit excellent mechanical, chemical, electrical and optical properties^1^. To utilize these enhanced properties of nanosuspensions, two-dimensional and three-dimensional structures^2^, such as thin films and discrete arrays, have to be manufactured. Direct printing of semiconductor materials using electrospray has the potential to significantly lower the cost of producing semiconductor devices compared to the conventional multistep method of photolithography and chemical processing. Two major steps in the new electrohydrodynamics (EHD) method are printing and solidification^3^.

The printing or deposition of the semiconductor materials requires a steady supply of a fixed amount of nanosuspension, which is achieved by driving the suspension through a nozzle using a piezoelectric device^4, 5^. EHD is another promising technique to achieve sub-micrometer resolution in jet printing and also eliminate the clogging problem compared to conventional nozzle-based inkjet technologies^6^. These advantages are due to the unique process physics in EHD for producing droplets of diameters smaller than the orifice of the nozzle, since the droplets can be formed and ejected from the apex of a conical meniscus instead of the tip of the nozzle. To implement this technique in practice, however, the process physics need to be understood and the technical challenges have to be overcome. Some of these problems are difficulties in preparing the nanosuspensions, uniformity and stability of the suspensions, optimizing their thermomechanical and electrical properties, nonuniformities in the printed microstructures due to coffee-ring effects, and early stages of research in semiconductor printing.

A nanoelectrospray laser deposition technique provides a convenient way of producing two-dimensional and three-dimensional structures. In this technique, nanoparticles are dispersed in water with an appropriate surfactant, which readily produces a viscous suspension called aqueous ink, and this nanosuspension can become increasingly viscous at high concentrations^7–9^. Deposition of nanoparticles using this ink in nanoelectrosprays can promote the fabrication of a variety of energy and electronic devices such as conformal solar cells, sensors, and actuators. This novel nanoparticle dispensing technique can also be used to fabricate masks for nanolithography, nanopillar arrays for photonic crystals, and nanodot arrays for plasmonic surfaces. Electric field-driven microdroplets can be used for thin film deposition^10, 11^, drop-on-demand printing^12^, and microencapsulation^13–15^.

Electrospraying of semiconductor materials has been studied in recent years for depositing thin films, such as copper-indium-diselenide films for solar cell^16^ and ZnO-based thin film transistors^17^. However, the effect of various properties of the nanosuspensions, such as viscosity, surface tension and electrical conductivity, on the mode of droplet formation needs to be studied to understand the stable operation of the EHD process for practical applications. This study focuses on the conical meniscus regime that appears following the dripping mode and generates the microdripping and oscillating microdripping modes at low flow rates. The objective of this work is to delineate the transition of these modes based on a single dimensionless parameter. This is achieved by studying several nanosuspensions at low viscosity and high electrical conductivity.

## Results

The nanoelectrospray generates microdroplets carrying nanoparticles at desired concentrations and deposits a wet layer on the substrate, which can be subsequently evaporated to produce thin films or discrete array of nanodots. In electrosprays, the detachment of droplets from the tip of a capillary tube occurs due to the balance of the electrodynamic and surface tension forces acting on the fluid. An electrostatic field between the tube and an electrode in the vicinity of the tube alters the mode of detaching the droplets, resulting in different fluid behavior such as dripping, microdripping, spindle, multispindle, and cone-jet spray mode^18–21^. Thus, the electro-capillary interaction provides an additional mechanism to generate different modes for the fluid dynamic response of the droplets.

The electrical conductivity and the flow rate of the liquid affect the droplet diameter^22–26^. This is due to the fact the electrical conductivity of the nanosuspension affects the cone geometry by inducing electrical tangential stresses^27, 28^. The presence of semiconductor nanoparticles inside the droplets, particularly the electrical conductivity of the nanomaterials, will influence the transition of the microdroplet modes. This is because the polarizable materials can interact with the electrostatic field more energetically than the aqueous liquid medium itself. In addition, the viscosity affects the shape of the cone by resisting its deformation and thus promotes a stable cone-jet spray mode. A fluid of higher viscosity also has the additional advantage of decreasing the jet diameter^29, 30^. The nanosuspensions used in this study and their properties are listed in Table 1. These liquids were selected for their high conductivity and low viscosity but with a reasonable variation in surface tension. The Reynolds number is calculated as *Re* = *ρu*_*c*_ *R*_*i*_ */μ*, where, *ρ*, *u*_*c*_, *R*_*i*_, and *μ* are the density of the fluid, the characteristic velocity (supplementary information file), the inner radius of the capillary tube at the tip, and the viscosity of the fluid. Experiments were carried out at low Reynolds numbers to investigate the modes of nanosuspensions in laminar electrospray of droplets.

**Table 1:** Physical properties of nanoparticle solutions in DI water.

Liquid	Surfactant molar concentration (10^−3^ M)	Dispersant Concentration (g/L)	Density ρ (g/ml)	Viscosity µ (10^−3^ Pa.s)	Conductivity σ (10^−4^ S/m)	Surface Tension γ (10^−3^ N/m)	Droplet radius at E = 0 r_o_ (mm)
SDS solution (6 mM)	6	0	1.0	1.13	1110.4	44.6	1.21
Si, 2 wt% in H_2_O	6	0.4	1.010	1.48	991.6	46.7	1.18
Si, 5 wt% in H_2_O	6	0.4	1.033	1.82	1391.6	48.2	1.18
Si, 10 wt% in H_2_O	6	0.4	1.060	2.72	1411.2	47.1	1.17
SiC, 2 wt% in H_2_O	8	1.0	1.051	1.56	785.4	37.7	1.47
SiC, 5 wt% in H_2_O	8	1.0	1.127	4.79	846.8	38.5	1.19
ZnO, 10 wt% in H_2_O	+	+	1.103	3.30	464.2	55.5	1.26

Note: Si and SiC nanoparticles are 30 nm in diameter and ZnO nanoparticles are 70 nm in diameter. All nanosuspensions are in water. (+) obtained from commercial suspension (Alfa aeser, product number 45012).

**Figure 1:**
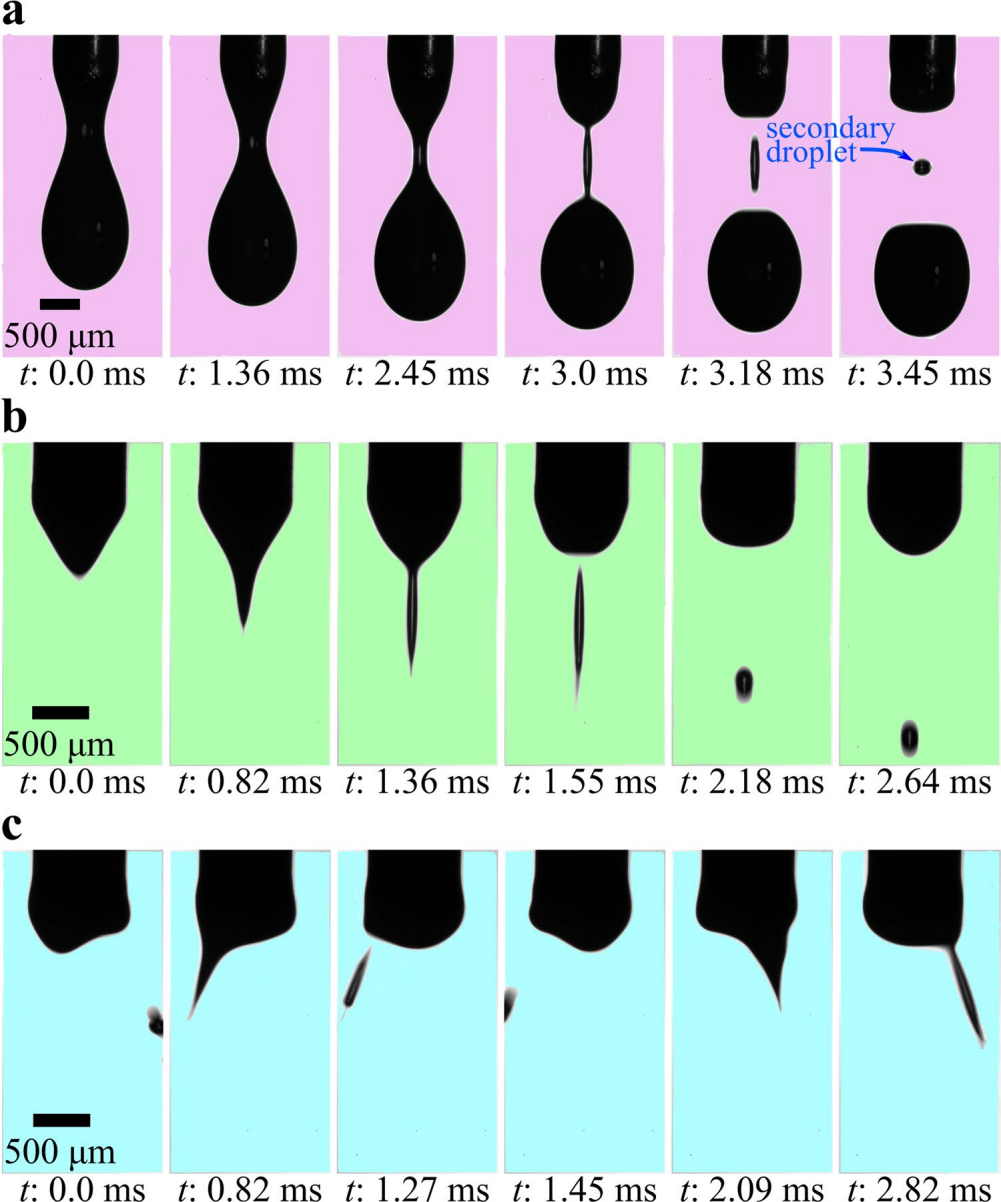
Droplet formation from the capillary tube under the action of an electric field. (**a**) Dripping mode of Si, 5 wt% in H_2_O (H_1_ = 4 mm, ϕ_o_ = 2500 V). The main droplet is formed along with a secondary droplet. (**b**) Microdripping mode of Si, 5 wt% in H_2_O (H_1_ = 4 mm, ϕ_o_ = 4000 V). Uniform monodisperse drops are generated from the conical meniscus. (**c**) Oscillating microdripping mode of Si, 5 wt% in H_2_O (H_1_ = 4 mm, ϕ_o_ = 5000 V). The conical meniscus oscillates in a plane ejecting drops in two directions.

Different electrospray modes, such as dripping, microdripping, and oscillating microdripping were observed, as shown in Fig. 1. In all modes, the gravity and electric fields stretch the meniscus downward while the surface tension force and viscous force oppose the stretching. In the dripping mode (Fig. 1a), the droplets are formed from a hemispherical or ellipsoidal meniscus at the tip of the capillary tube, whereas the field-induced stretching of the fluid creates a conical meniscus in the other modes (Fig. 1b,c). The electric field stretches these cones to a critical dimension and pinches off the elongated liquid filament that subsequently deforms into a spherical droplet to achieve a state of minimum surface energy. In the dripping mode, a small secondary droplet can be observed above each large droplet (Fig. 1a). This tiny droplet is formed due to the stretching at the neck between the meniscus and the large droplet. The neck detaches as an isolated narrow liquid filament that eventually reshapes into the secondary spherical droplet. In each mode, the meniscus goes through a sequence of deformation to produce the droplets as shown in Fig. 1, and the sequence repeats after the detachment of the droplets. As a reference time for this type of periodic sequence, t = 0 refers to the time at which the first meniscus shape is formed in the dripping mode (Fig. 1a), microdripping mode (Fig. 1b), and oscillating microdripping mode (Fig. 1c).

In the microdripping mode, the conical meniscus is axisymmetric with the tip of the cone on the axis of the capillary tube, and spherical droplets are vertically ejected toward the substrate through the annular copper disk electrode. A stable mechanism is established in this mode, which produces a steady stream of uniform microdroplets (Fig. 1b) that drop in the vertically downward direction. This mode is, therefore, suitable for depositing discrete arrays of nanodot, which is a cluster of nanoparticles, to produce advanced optoelectronic and microelectronic devices at a high speed.

As the voltage is increased, the cone-tip oscillates in a plane around its axisymmetric position like a pendulum (Fig. 1c), and microdroplets are ejected from the cone-tip when the tip is at the circumference of the meniscus. The frequency of these oscillations varies from 150 Hz for ZnO, 10 wt% suspension to 250 Hz for Si, 2 wt% suspension. Consequently, the droplets are obliquely directed toward the substrate in two different directions. At low electric fields up to the microdripping mode, the electric energy is sufficiently low and the liquid molecules and nanoparticles in the meniscus are polarized in a regular pattern aligned in the vertical direction, resulting in a stable symmetric meniscus that produces a steady vertical stream of droplets. As the electric field increases, the polarized energy in the liquid molecules and nanoparticles increases and these polarized states interact with each other, resulting in an unstable asymmetric meniscus that creates the oscillating microdripping mode.

**Figure 2:**
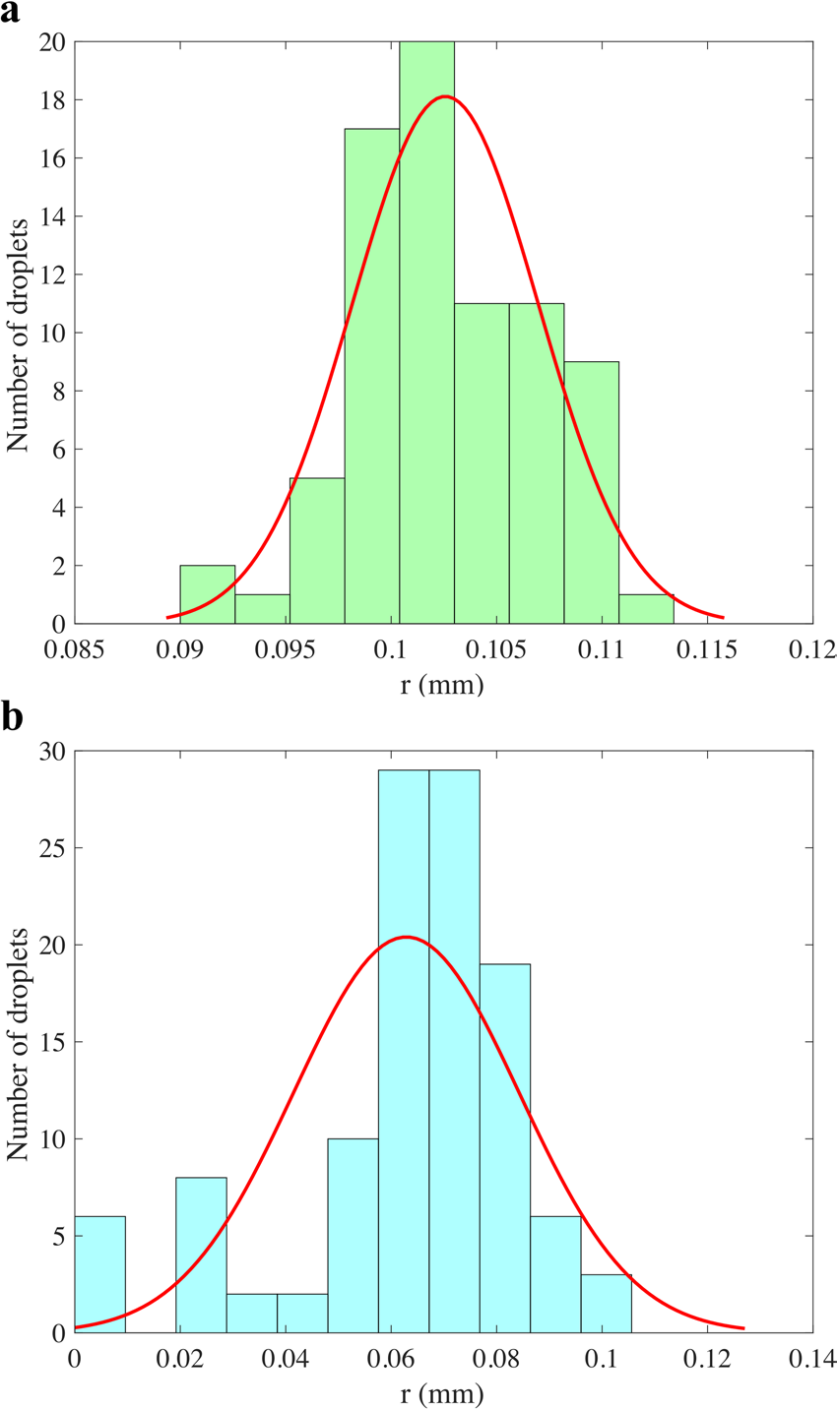
Example of size distribution of ejected droplets. (**a**) Histogram of the radius of ejected droplets for SiC, 5 wt% in H_2_O in microdripping mode (ϕ_0_ = 3000 V and H_1_ = 2 mm). (**b**) Histogram of the radius of ejected droplets for SiC, 5 wt% in H_2_O in oscillating microdripping mode (ϕ_0_ = 4500 V and H_1_ = 2 mm). The modes are color coded corresponding to Fig. 1.

**Figure 3:**
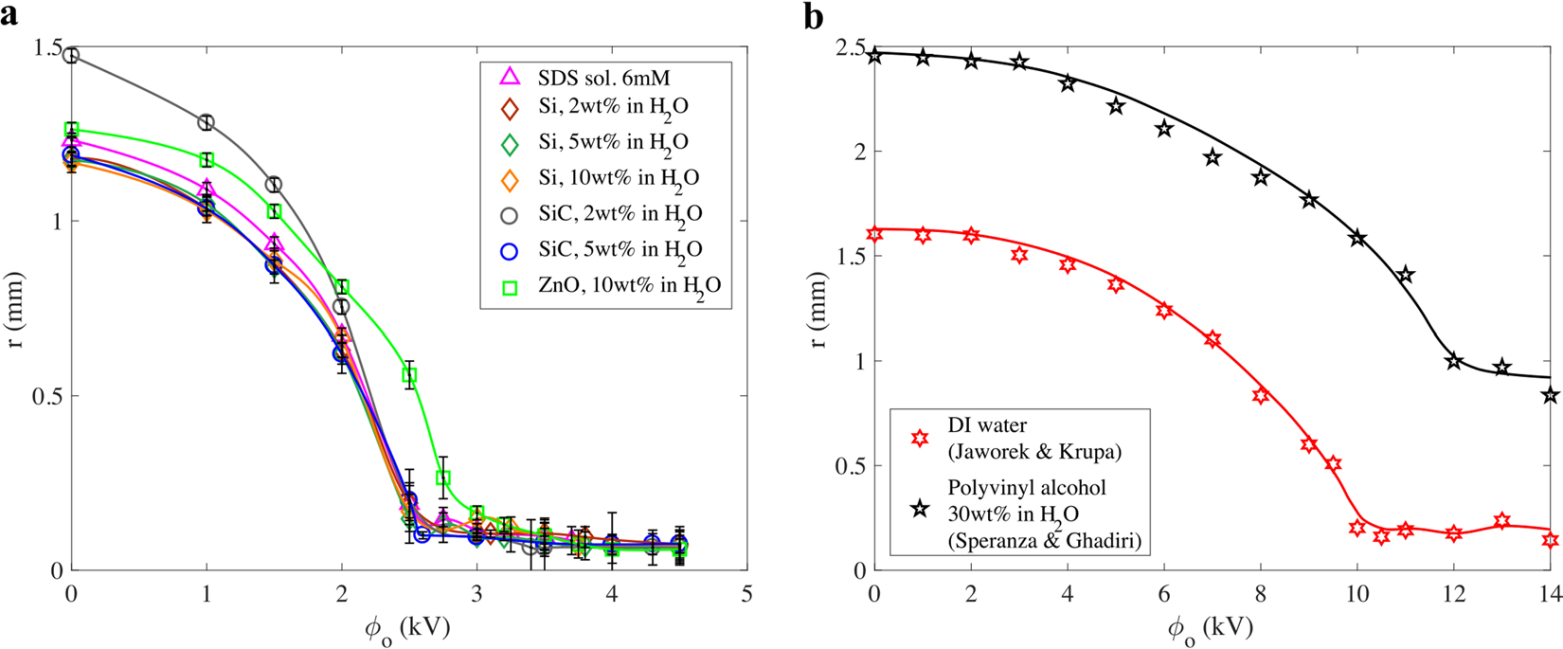
Droplet size vs applied electrical voltage. (**a**) Markers represent the average droplet radius as a function of the applied voltage between the capillary tube and the earthed electrodes. Error bars show the uncertainty of the measurements with a confidence interval of 95%. Solid curves are produced by curve fitting the experimental results. (**b**) Other investigators’ data. Jaworek & Krupa’s results^19^ (red stars) are adapted from their Fig. 3a. Speranza & Ghadiri’s results^31^ (black stars) correspond to their electrode configuration C. Solid curves are produced by curve fitting the experimental data.

Figure 2 shows the size distribution of ejected droplets for the nanosuspension of 5 wt% SiC in H_2_O in the microdripping (Fig. 2a) and the oscillating microdripping (Fig. 2b) modes respectively. It can be seen that the average radius of the droplets, which is computed as the mean value, is larger for the microdripping mode (102 μm) than for the oscillating microdripping mode (63 μm). However, the microdroplets are produced with a very narrow standard deviation of 9 μm in the former mode compared to the relatively large standard deviation of 43 μm in the latter mode. The average radii of droplets are shown in Fig. 3a with the corresponding uncertainty in the measurements for different nanosuspensions and different voltages up to 5 kV applied to the capillary tube. Two sets of data in the literature^19, 31^ are also provided in Fig. 3b. The average droplet size decreases as the applied voltage increases. An increase in the electric field due to increasing voltage causes breakage and subsequent fragmentation of the droplet when the electric field is sufficiently large, which causes a reduction in droplet radius. Although the dropsize decreases with increasing voltage, the size distribution will broaden as shown in Fig. 2 and the electric repulsion between ejected droplets will become significant. The region between the dripping and microdripping modes is a mixed mode regime where either large or small diameter is possible depending on the nanosuspensions. However, for all suspensions, microdripping occurs after this mixed mode, which highlights the importance of knowing the characteristic voltage at which microdripping occurs.

**Figure 4:**
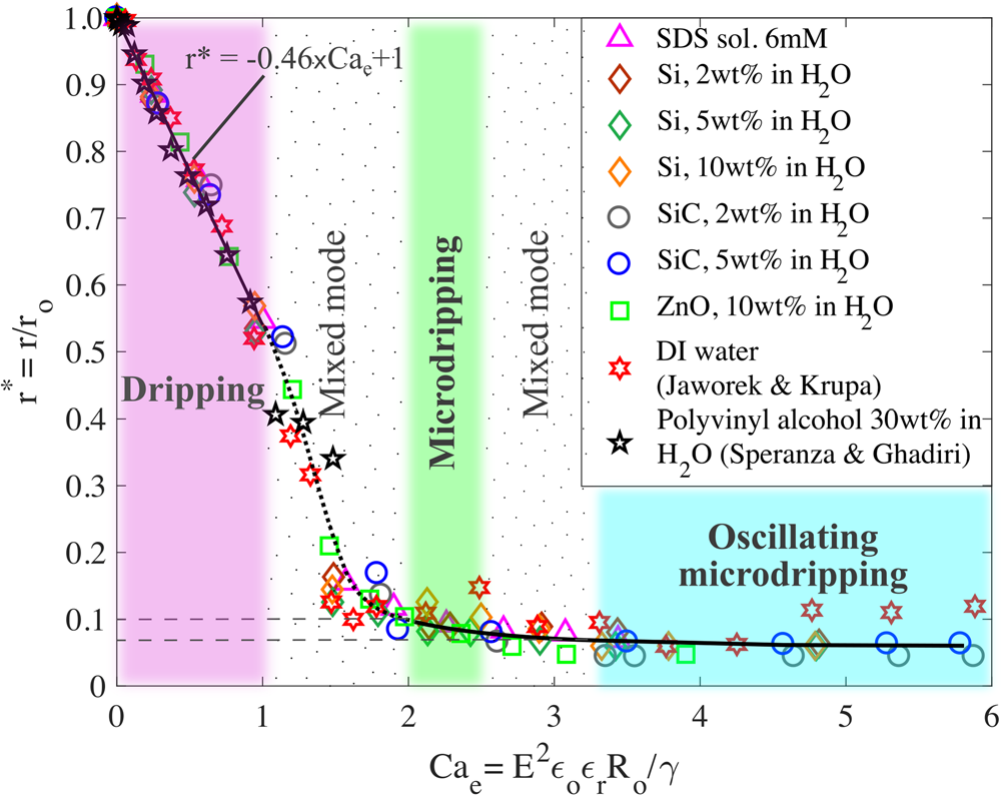
Dimensionless size of droplets vs electric capillary number. Results from Fig. 3a,b are nondimensionalized with respect to the droplet radius in the absence of electric field and displayed as a function of the electric capillary number. Transition between dripping and microdripping modes occurs at *Ca*_*e*_ ≈ 1.0 and the transition between microdripping and oscillating microdripping modes occurs at *Ca*_*e*_ ≈ 2.5. The modes are color coded corresponding to Fig. 1.

It should be noted in Fig. 3a that the dropsize for different suspensions spreads over a wide band as a function of voltage. The spread in the data can be collapsed into a single curve as a function of a nondimensional parameter called the electric capillary number, *Ca*_*e*_^32, 33^. The data in both Fig. 3a,b are fitted into a single curve in Fig. 4, i.e., *Ca*_*e*_ = E^2^ *ϵ*_o_ *ϵ*_r_R_0_/γ, where E, *ϵ*_o_, *ϵ*_r_, R_0_ and γ are the externally applied electric field, electrical permittivity of vacuum, characteristic relative permittivity for each aqueous suspension, outer radius of the capillary tube, and the surface tension of the suspension respectively. The characteristic relative permittivity is defined as *ϵ*_r_ = 1 + σ/*ϵ*_o_ω based on the Lorentz model for the interaction of electromagnetic waves in dielectric materials^34^, where σ is the electrical conductivity of each aqueous suspension and ω is a characteristic frequency taken as c/L where c and L are the speed of light in air and the distance from the center of the capillary tip to the inner edge of an annular disk electrode, i.e., L = $\sqrt{{H}_{1}^{2}+{R}_{id}^{2}}$, where *R*_*id*_ and *H*_*1*_ are the inner radius of the disk and the distance between the capillary tube and disk respectively.

The expression for *E* in *Ca*_*e*_ was determined as *E*=*E*_*1*_+*E*_*2*_ for the three-electrode system of this study, where *E*_*1*_ is the electrostatic field between the capillary tube and the disk, and *E*_*2*_ is the electrostatic field between the tube and the substrate. The tube was connected to the positive polarity, and the disk and substrate were connected to a ground terminal. *E*_*1*_ was determined using *E*_1_ = $\sqrt{2}{\varphi }_{o}/{R}_{0}\,\mathrm{ln}(4{H}_{1}/{R}_{0})$, and *E*_*2*_ using *E*_2_ = $\sqrt{2}{\varphi }_{o}/{R}_{0}\,\mathrm{ln}(4{H}_{2}/{R}_{0})$, where ϕ_o_ is the electric voltage and *H*_*2*_ is the distance from the center of the tip of the capillary tube to the substrate. Note that Jones and Thong^35^ derived the expression of the electrostatic field for a two-electrode system consisting of a thin cylinder with rounded tip of positive polarity and a large semi-infinite solid as the ground terminal and, therefore, *E* = *E*_*2*_ for the droplets produced by the two-electrode system^19^. The electric capillary number varies in the range 0 ≤ *Ca*_*e*_ ≤ 6 for the cases analyzed in this study.

The droplet radii of different types of suspension are found to follow a unique trend as a function of the electric capillary number (Fig. 4) for a given flow rate of the suspension through the capillary tube. The effect of inertial force is neglected since the flow rate is very low and the densities of the aqueous suspensions are of the same order (Table 1). Fig. 4 clearly classifies the transition of droplet modes into two basic regimes: ellipsoidal and conical meniscus regimes based on the shape of the meniscus at the tip of the capillary tube. While ellipsoidal meniscus causes the dripping mode, the conical meniscus produces microdripping and oscillating microdripping modes depending on the electric capillary number.

The mechanism of droplet formation in the EHD process depends on the electrical and gravity forces, which tend to detach the droplets from the meniscus, and the surface tension and viscous forces that resist the detachment of the droplets. The relative influence of these forces on the droplet size is determined by the nondimensional parameters: Electric capillary number, Capillary number (*Ca* = μ*u*_*c*_ */γ*) and Bond number (*Bo* = *Δρgr*_*o*_^2^ */γ*), each appearing as a ratio of the respective force to the surface tension force. In the dripping mode, the capillary number is typically 0.01 based on *u*_*c*_ ≈ 0.25 m/s (Supplementary Table S1 in the supplementary file), which indicates that the viscous effects are negligible in this mode for the fluids tested in this study. Since Bo contains *r*_*0*_, which is the radius of droplets formed by the gravity, surface tension and viscous forces in the absence of any applied voltage, the effect of Bo is implicitly considered in the nondimensional radius r*. The nondimensional radius of droplets, r*, is modeled as a linear function of *Ca*_*e*_ with slope b in the dripping mode, such as r* = 1+*bCa*_*e*_ which satisfies the condition *r* = *r*_*o*_ when the electric field E or the electric capillary number *Ca*_*e*_ is zero. Since the maximum value of r* is 1, the slope b will be negative which is empirically found to be b = −0.46 from Fig. 4 in the range 0 ≤ Ca_e_ ≤ 1. Consequently, the expression for r* is, r* = 1–0.46*Ca*_*e*_ in this mode.

All the liquids of this study are observed to be in the dripping mode up to *Ca*_*e*_ = 1 at which point the electrical and surface tension forces are exactly balanced. Also at this point, the radius r* reduces from 1 to 0.5 which corresponds to approximately 1 to 0.5 mm. This mode is, however, observed in the region slightly after *Ca*_*e*_ = 1 for certain suspensions such as SiC 2 wt%, SiC 5 wt%, and ZnO 10 wt%, and a new mode called the microdripping mode appears slightly before *Ca*_*e*_ = 2 for the suspensions Si 2 wt%, Si 5 wt%, and Si 10 wt%. This region of 1 ≤ *Ca*_*e*_ ≤ 2 is designated as the ‘mixed mode’ region in this study since both the dripping and microdripping modes coexist in this region with the droplet radius varying approximately from 0.5 mm to 0.1 mm. The scaling law of Fig. 4 also conforms to other investigators’ data using low conductivity liquids such as DI water without any suspension^19^ and 30 wt% polyvinyl alcohol in water^31^.

**Figure 5:**
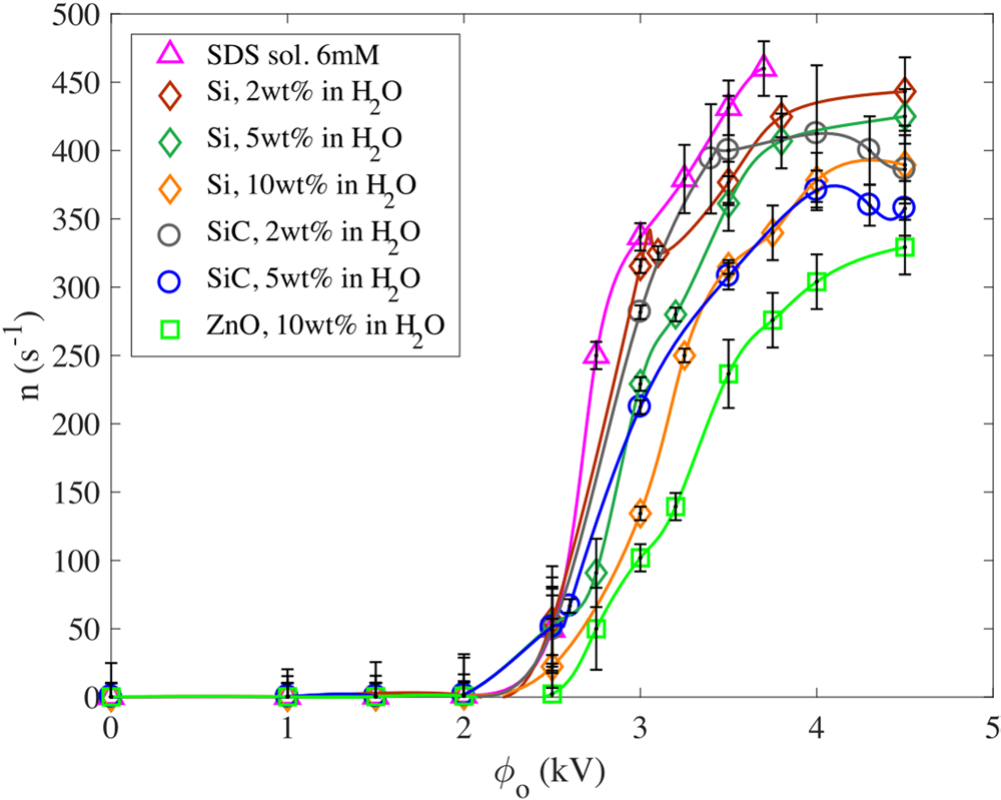
Meniscus frequency vs applied electric voltage. Markers represent the average meniscus frequency as a function of the applied voltage between the capillary tube and the earthed electrodes. Error bars show the uncertainty of the measurements with a confidence interval of 95%. Solid curves are produced by curve fitting the experimental data.

**Figure 6:**
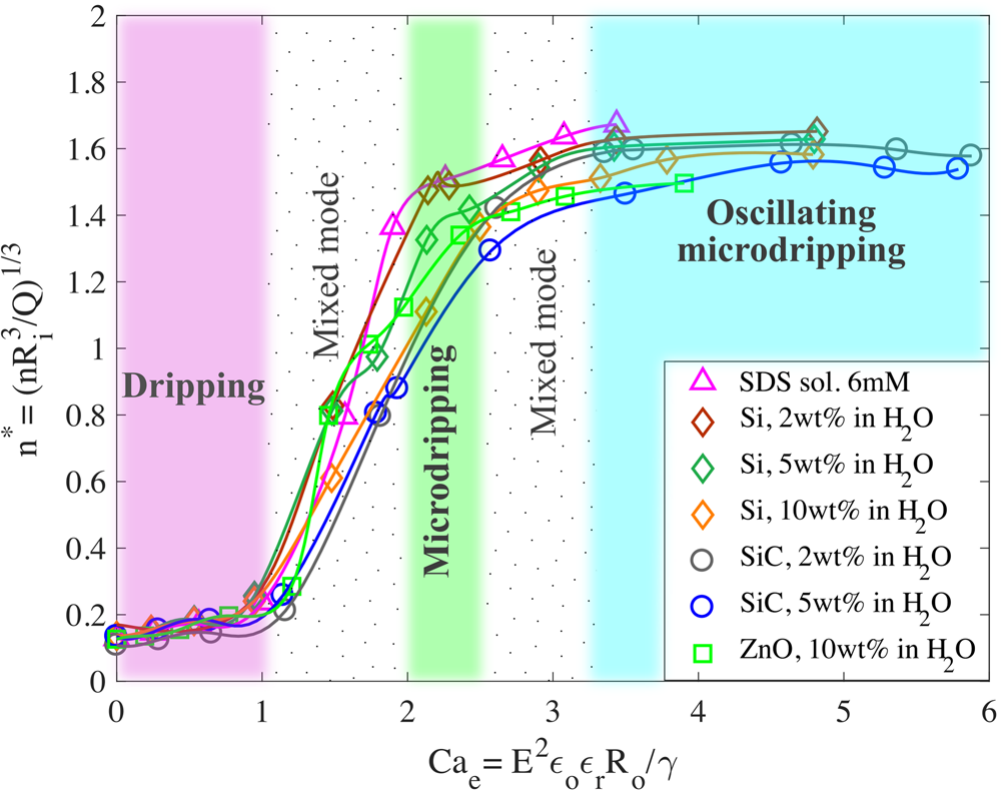
Dimensionless meniscus frequency vs electric capillary number. The frequency changes abruptly at the transition between dripping and microdripping mode. Fluids with high capillary number tend to have lower frequency at a given electric capillary number due to viscous effects. The modes are color coded corresponding to Fig. 1.

Immediately after the mixed mode region, the conical meniscus regime is observed with the microdripping mode occurring first, followed by oscillating microdripping mode with an increase in *Ca*_*e*_. In addition, all the suspensions of this study exhibited the microdripping mode over a narrow range 2 ≤ *Ca*_*e*_ ≤ 2.5. The droplet radius varies from 0.1*r*_*o*_ to 0.07*r*_*o*_ corresponding to approximately 100 μm to 70 μm, which indicates that the dropsize is nearly constant. However, the velocity of the droplets is large and the capillary number is larger than in the dripping mode (Supplementary Table S1 in the supplementary file) and, therefore, the viscous effects are expected to be significant in the microdripping mode. Although the viscous effects are not perceptible for r* in Fig. 4, they are found to influence the droplet frequency as shown in Figs 5 and 6. In the region 2.5 ≤ *Ca*_*e*_ ≤ 3.3, the microdripping mode is observed at slightly after *Ca*_*e*_ = 2.5 for certain suspensions, such as SiC 2 wt%, SiC 5 wt%, and ZnO 10 wt%, and a new mode called the oscillating microdripping mode appears slightly before *Ca*_*e*_ = 3.3 for the suspensions Si 2 wt%, Si 5 wt%, and Si 10 wt%. This range of *Ca*_*e*_ is, therefore, designated as the second mixed mode region where either microdripping or oscillating microdripping mode can be observed depending on the suspension.

The transition between any two modes is based on the shape of the meniscus at the tip of the capillary tube, particularly the ellipsoidal and conical shapes observed during the experiments in this study (Fig. 1). The ellipsoidal meniscus causes the dripping mode in which the gravity and surface tension forces are dominant and the effect of the electric filed is relatively low. The conical meniscus produces microdripping and oscillating microdripping modes in which the electric field and surface tension forces are dominant and the effect of the gravity force is relatively low. Since the size of ejected droplets is significantly affected by the shape of the meniscus, the transition to microdripping is noticeable by a change in the slope of the r* versus *Ca*_*e*_ data. This type of change in the slope is gradual in Fig. 4 at the transition boundaries of the microdripping and oscillating microdripping modes since the variation in the radii of the droplets is very small between these modes. However, the transition boundaries between the microdripping, second mixed mode and oscillating microdripping regions were discerned by observing the changes in the shape of the meniscus as presented in Fig. 1. Based on the experimental observation, the oscillating microdripping mode was observed beyond *Ca*_*e*_ ≈ 3.3 for all the nanosuspensions tested in this study. The mechanism of mode transition is a complex physical process involving various electrohydrodynamic forces and the meniscus instability.

This type of mode-shifting at higher voltages can be attributed to two effects: (i) The electrostatic field interacts with the materials of low and high conductivities, such as dielectrics, polar surfactant molecules, n-type and p-type semiconductors and metals, differently due to their difference in polarizability. The distributions of the polarized nanoparticles and polar liquid molecules are expected to be asymmetric in the azimuthal direction since the nanoparticles and the molecules need not be distributed symmetrically in the original suspension. The effect of this asymmetric distribution becomes significant when the electric field increases, giving rise to asymmetric force components to induce the oscillating microdripping mode; and (ii) The repulsive Coulomb force between charged particles produces tangential stress on the conical surface of the meniscus.

More polarization energy is stored in the meniscus at higher voltages and, therefore, smaller droplets are formed so that the total mechanical energy, i.e., the surface formation energy, of all the droplets balances the electrical energy. Figure 4 also shows that the droplet size is nearly constant at higher values of *Ca*_*e*_, which indicates that the droplets have reached a state at which maximum surface energy is stored in each droplet. The critical radius in this study refers to the radius at which the microdripping mode begins. From Fig. 4, this critical radius is r* = 0.1 that occurs at *Ca*_*e*_ ~ 2 for all the semiconductor suspensions.

An increase in the applied voltage will increase the droplet frequency *n*, i.e., the number of droplets produced per unit time, at higher electric fields as evident in Fig. 5. The droplet frequency, which is plotted as a function of the applied voltage in this figure, spreads over a wide band for a given voltage. To examine the effect of *Ca*_*e*_ on the droplet frequency bandwidth, nondimensional droplet frequency is presented in Fig. 6 as a function of *Ca*_*e*_. Since the viscosity as well as other parameters such as the flow rate and density of liquid affect the droplet frequency, the capillary number (*Ca*) is used to parametrize the data (Supplementary Table S1). The nondimensional frequency is defined as n* = ${(n{R}_{i}^{3}/Q)}^{1/3}$, where Q is the volumetric flow rate. In Fig. 6, for a given *Ca*_*e*_, the frequency decreases as *Ca* increases. The 5 wt% SiC nanoparticle suspension, which has the highest *Ca* yields the lowest frequency. On the other hand, the 6 mM SDS solution, which has the lowest *Ca* yields the highest frequency. This trend in the frequency is due to an increase in viscosity (i.e. increase in the nanoparticle concentration) that dampens the growth of perturbations within the cone^36^. Thus viscosity delays the breakage of the meniscus in the microdripping and oscillating microdripping modes due to strong bonding of the liquid molecules and nanoparticles. However, viscosity does not affect the droplet size significantly in the dripping mode as shown in Fig. 4 where the data are plotted as a function of *Ca*_*e*_ which is independent of viscosity. Note that *Ca* is about 60 to 80% less in the dripping mode than in the microdripping mode (Supplementary Table S1), which indicates that the viscous effects are less significant in the dripping mode. Since the capillary numbers are large in the microdripping mode (*Ca*_*e*_ > 2.0) due to high velocity within the meniscus (Supplementary Table S1), the viscous effects become more significant in affecting the dynamics of the conical meniscus.

## Discussion

The modes of nanosuspensions containing semiconductor nanoparticles have been studied in laminar electrospray of droplets. A suitable microdripping mode has been observed, which can be utilized for depositing monodisperse microdroplets by the electrospray technique. This will allow an innovative approach for manufacturing energy, optical and electronic devices. A regime map is provided for identifying the limits to operate the EHD process under the microdripping mode to achieve uniform monodisperse microdroplets. An increase in nanoparticle concentration increases the liquid viscosity, which dampens the growth of perturbation and delays the jet breakup in the microdripping mode. However, viscosity does not affect the drop size in the dripping mode. For the given flow rate used in this study, the transition between ellipsoidal and conical meniscus regimes is found to occur at *Ca*_*e*_ ≈ 1.0. The dripping mode occurs for *Ca*_*e*_ < 1 when the droplet size strongly depends on the electric capillary number (r* = −*0*.*46Ca*_*e*_ + *1*) compared to the droplets of nearly constant radius at r* = 0.1 and r* = 0.07 generated in microdripping and oscillating microdripping modes. Thus, the average droplet size can be determined using these results if the variables that define the electric capillary number and the radius of the droplet (*r*_*0*_) in the absence of electric field are known. Electrical conductivity affects the transition between microdripping and oscillating microdripping modes. The onset of oscillating microdripping mode occurs first for suspensions with higher conductivity. Ejection of uniform microdroplets in the microdripping mode is observed in the range 2.0 ≤ *Ca*_*e*_ ≤ 2.5 and oscillating microdripping mode is reached at *Ca*_*e*_ ≥ 3.3 for all the nanoparticle suspensions of this study.

## Methods

### Solution preparation

Experiments were conducted in this study using aqueous colloidal suspension of a variety of semiconductor nanoparticles as listed in Table 1. The suspensions were prepared by dispersing the nanoparticles in de-ionized (DI) water with a surfactant and dispersant, and sonicating the mixture in a cup-and-horn-type ultrasonicator for one hour. The surfactant was sodium dodecyl sulfate (SDS) with concentration from 1.7 to 2.3 g/L (6 × 10^−3^ to 8 × 10^−3^ M) in DI water and sodium salt of poly-naphthalene sulfonic acid was used as anionic dispersing agent of concentration varying from 0.4 to 1.0 g/L of DI water. To examine the effect of nanoparticles on the microdroplet mode, another solution of just the DI water and surfactant was prepared so that the surface tensions of this solution and the nanoparticle suspension were the same, and this control solution is listed as SDS solution (6 mM) in Table 1.

### Experimental Setup

**Figure 7:**
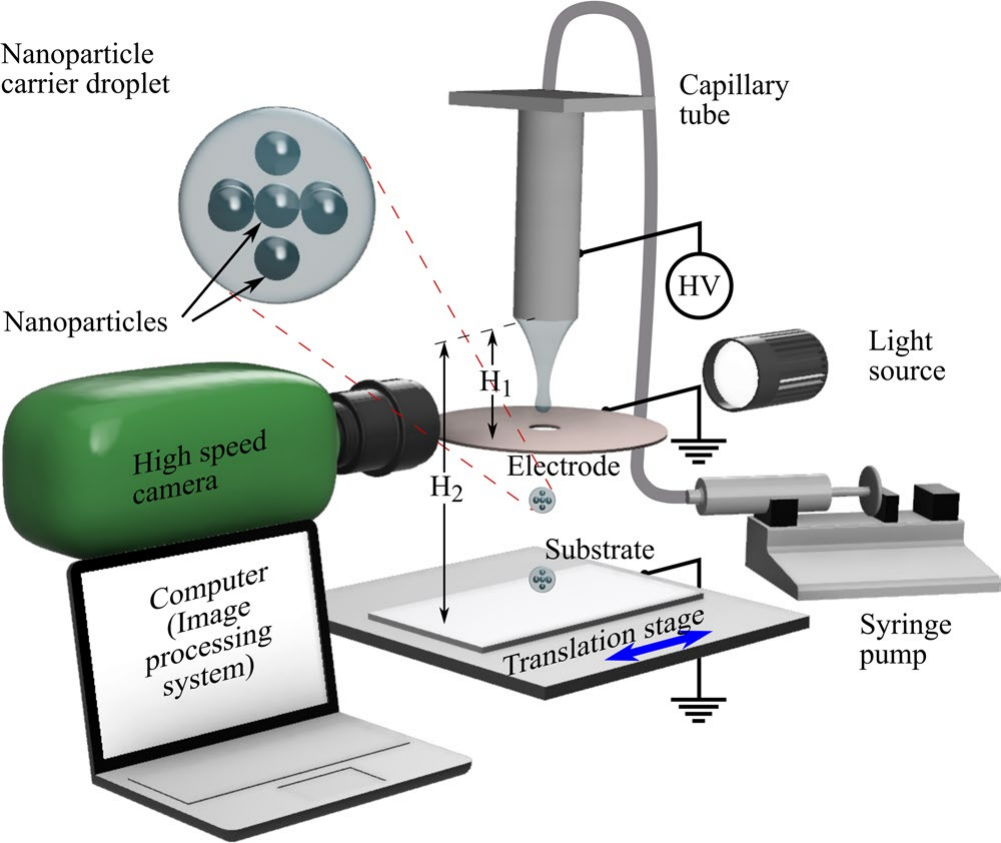
Schematic representation of experimental setup. The electrostatic field is created between the capillary tube connected to a positive high voltage source and the earthed disk electrode and moving substrate. The high speed camera and light source are aligned with the capillary and droplet ejection from the liquid meniscus.

The viscosity, surface tension and electrical conductivity of the resulting suspensions were experimentally obtained using a Brookfield DVII + Pro viscometer, a SITA Dynotester bubble pressure tensiometer, and an Omega handheld conductivity tester, respectively. The sonicated liquid was supplied at a flow rate of Q = 1.67 mm^3^/s to a stainless steel capillary tube of inner and outer diameters 0.51 and 0.82 mm, respectively. An electrostatic field was created by applying a direct current positive voltage to the capillary, and an annular copper disk and the substrate as the ground terminal as shown in Fig. 7. The inner and outer diameters and the thickness of the disk were 4.5, 38 and 0.5 mm, respectively, and the disk was coaxially situated 2 mm (H_1_) below the tip of the capillary tube (unless otherwise specified) and 70 mm (H_2_–H_1_) above the substrate surface. The applied voltage was varied to generate droplets of different diameters, and their sizes were recorded using high speed photography (Phantom V12.1 camera). All experiments were carried out at 25 ± 1 °C and the system was allowed at least 30 min to reach the steady state of droplet formation before video recording any data.

### Image processing of droplets and meniscus contours

For the measurement of the droplet diameter and the frequency of the meniscus, videos of 512 × 1024 pixels (spatial resolution of 5.5 μm/pixel) were recorded at different frame rates depending on the mode being recorded. The dripping, microdripping, and oscillating microdripping modes were recorded at 2000 fps, 8000 fps, and 11000 fps respectively. In general, 670 frames were captured at each applied voltage. Thus, each video records the ejection of several droplets. In addition, the entire set of experiments (i.e., 0 ≤ ϕ_0_ ≤ 5000 V) was repeated at least three times for each semiconductor nanosuspension including the SDS solution.

Using the video data, the volume of the droplets was calculated by image processing. Since the shape of the droplets was not perfectly spherical and varied with time during their free fall, a Riemann sum, i.e., the sum of slices of 1 pixel each, was used to compute the volume, *V* = $\sum _{i=0}^{n}\pi {r}_{i}^{2}$, under the assumption that the falling droplet is axisymmetric at all instances. Here, *r*_*i*_ is the radius of the *i* ^th^ slice. The radius of each droplet was then determined as *r* = $\sqrt[3]{3V/4\pi }$. Finally, the average radius of the droplet was calculated as the mean value and its standard deviation was used to compute the uncertainty in the measurements.

**Figure 8:**
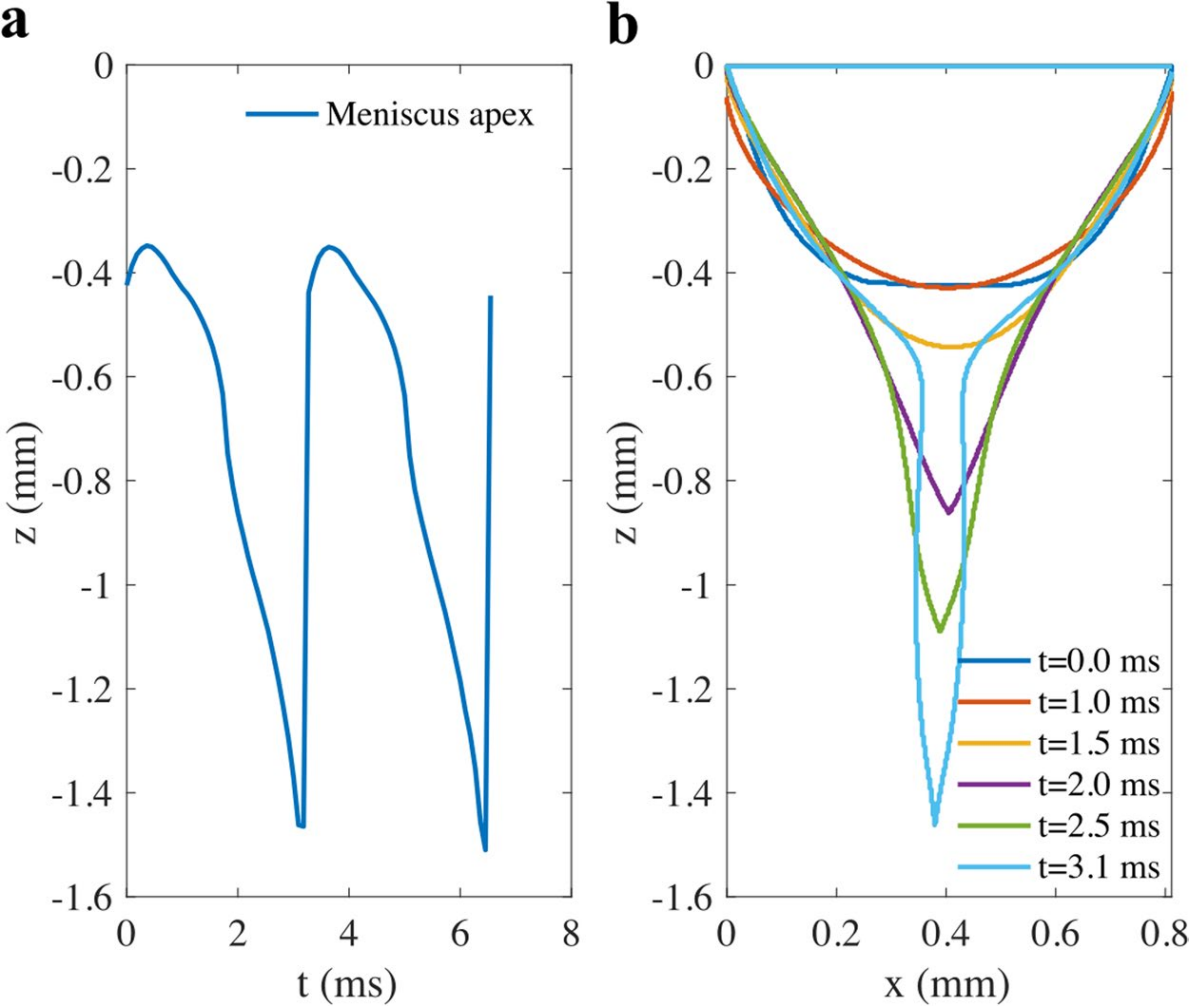
Image processing of meniscus contour. (**a**) Meniscus apex location as a function of time of electrospray operating in microdripping mode (Si, 5 wt% in H_2_O, H_1_ = 4 mm, ϕ_o_ = 4000 V). Two periods are shown which consist in the formation of two monodisperse microdroplets at t = 3.1 ms and t = 6.4 ms. (**b**) Sequence of the meniscus contour of microdripping mode corresponding to case used in (**a**).

Also the frequency at which the liquid meniscus oscillates at the tip of the capillary tube was determined using the video image that recorded the growth of the meniscus apex as a function of time as shown in Fig. 8. The Fig. 8a represents the periodic stretching and contraction of the apex of the conical meniscus in the microdripping mode with the apex stretching to a fixed length at the time interval of approximately 3.2 ms. Figure 8b shows the deformation of the meniscus with the apex moving vertically at a fairly high speed of nearly 0.6 m/s, indicating that microdroplets can be dispensed at a high rate to deliver nanoparticles for high speed manufacturing.

## Electronic supplementary material

Relevant parameters in electrosprays
