# Supplementary material for: Electrospray mode transition of microdroplets with semiconductor nanoparticle suspension
Source: Sci Rep. 2017 Jul 11;7:5144. doi: 10.1038/s41598-017-05175-6 (PMC5506035; doi:10.1038/s41598-017-05175-6)
Supplement: Supplementary file 1 — Relevant parameters in electrosprays [file 41598_2017_5175_MOESM1_ESM.doc]

**Electrospray mode transition of microdroplets with semiconductor nanoparticle suspension**

Eduardo Castillo-Orozco^†^, Aravinda Kar^††^, Ranganathan Kumar^†*^

**Supplementary information**

**Selection of non-dimensional frequency in Figure 6.**

By mass balance, the radius of the droplet may be approximated as , where *Q* and *n* are the volumetric flow rate and number of droplets ejected per unit time respectively. Thus,

$$r\sim\left(Q/n\right)^{1/3}$$

Then, $r_{n}^{*}=\frac{r}{R_{i}}\sim\left(Q/nR_{i}^{3}\right)^{1/3}$

where *R_i_* is the inner radius of the capillary tube and $1/r_{n}^{*}$ can be interpreted as the non-dimensional frequency of droplet ejection. Since the droplet frequency, n, increases as the droplet radius decreases, and *Ca_e_* increases, $1/r_{n}^{*}$ is plotted in Fig. 6 to show the increasing trend of the non-dimensional droplet frequency as a function of *Ca_e_*.

**Relevant parameters**

The characteristic velocity, *u_c_* can be determined by considering an energy balance between the kinetic and surface energy per unit volume.

$$\frac{1}{2}\rho u_{c}^{2}=\frac{\gamma}{r_{c}}$$

$$u_{c}=\left(\frac{2\gamma}{\rho r_{c}}\right)^{1/2}$$

where, *ρ*, *γ*, and *r_c_* are the density, surface tension, and the characteristic radius respectively. *r_c_* ~ *r_o_*, *r_c_* ~ 0.1*r_o_*, and *r_c_* ~ 0.07*r_o_* for dripping, microdripping, and oscillating microdripping mode respectively. Thus,

$$Ca=\frac{\mu u_{c}}{\gamma}$$

will have different values in the dripping and microdripping modes.

The Bond number and Reynolds number are defined as,

$$Bo=\frac{\Delta\rho gr_{o}^{2}}{\gamma}$$

$$\mathrm{Re}=\frac{\rho u_{c}R_{i}}{\mu}$$

where, *g*, *r_o_*, and *μ* are the gravitational acceleration, droplets radius in the absence of electric field, and viscosity of the fluid respectively. Supplementary table S1 shows the relevant parameters.

Supplementary Table S1: Relevant parameters for the three modes.

| **Liquid** |  |  |  | **Dripping** | | **Microdripping** | | **Oscillating microdripping** | |
| --- | --- | --- | --- | --- | --- | --- | --- | --- | --- |
|  | ***r_o_***  **(mm)** | ***Bo*** | ***Re*** | ***u_c_***  **(*m/s*)** | ***Ca***  **x10^-2^** | ***u_c_***  **(*m/s*)** | ***Ca***  **x10^-2^** | ***u_c_***  **(*m/s*)** | ***Ca***  **x10^-2^** |
| SDS solution (6mM) | 1.21 | 0.33 | 61.21 | 0.27 | 0.68 | 0.85 | 2.16 | 1.02 | 2.58 |
| Si, 2 wt% in H_2_O | 1.18 | 0.30 | 49.12 | 0.28 | 0.89 | 0.89 | 2.81 | 1.06 | 3.35 |
| Si, 5 wt% in H_2_O | 1.18 | 0.29 | 41.09 | 0.28 | 1.06 | 0.89 | 3.37 | 1.07 | 4.02 |
| Si, 10 wt% in H_2_O | 1.17 | 0.30 | 21.61 | 0.28 | 1.60 | 0.87 | 5.04 | 1.04 | 6.03 |
| SiC, 2 wt% in H_2_O | 1.47 | 0.59 | 38.29 | 0.22 | 0.91 | 0.70 | 2.88 | 0.83 | 3.44 |
| SiC, 5 wt% in H_2_O | 1.19 | 0.40 | 14.48 | 0.24 | 2.99 | 0.76 | 9.45 | 0.91 | 11.29 |
| ZnO, 10 wt% in H_2_O | 1.26 | 0.31 | 24.25 | 0.28 | 1.68 | 0.89 | 5.31 | 0.75 | 6.35 |
